# The Inspection of Veal*Read before the Philadelphia Society of Veterinary Medicine.

**Published:** 1892-02

**Authors:** C. H. Magill


### THE INSPECTION OF VEAL.*

By C. H. Magill, V. M. D.

At the last meeting of this Society the question of inspection of veal was brought up for discussion, as the city authorities have had some twenty or more dealers in veal placed under bail of $800 to appear at court for selling "monkey" veal (which is very young calf). Their weight ranged from 27 to 52 pounds. Two were found in the market on December 12 ; one only weighed 27 pounds, the other 32 pounds. A committee of three was appointed—Drs. Williams, Harger and myself—to make a report to the Society on inspection of veal, for the purpose of deciding at what age veal is fit for food. I have not been able to find anything concerning veal as a diet excepting in the "Text Book of Human Physiology," by Laudois & Sterling, third edition, page 405, under the heading of "Conditions of Diet for Health."

In an adequate diet not only, first, should the total quantity be sufficient and not more than sufficient ; but, second, the constituents should exist in proper proportions; third, be digestable; and fourth, the whole should be in good condition, wholesome and not adulterated with any substance prejudicial to health. With regard to the relative proportions of the various kinds of food which ought to be taken, experience has shown that the diet best suited for the body must contain 1 part of nitrogenous matter to 3½ parts, or at most 4½ parts of non-nitrogenous. Looking at ordinary food from this point of view, we see how far they correspond to this requirement, and how several substances may be combined to produce a satisfactory diet.

| | Nit. | Non-Nit. |
|---|---|---|
| Veal | 10. | 1. |
| Hare's flesh | 10. | 2. |
| Beef | 10. | 17. |
| Lentils | 10. | 21. |
| Beans | 10. | 22. |
| Peas | 10. | 23. |
| Mutton | 10. | 27. |
| Pork | 10. | 30. |
| Cow's milk | 10. | 30. |
| Human milk | 10. | 37. |
| Wheat flour | 10. | 46. |
| Oat meal | 10. | 50. |
| Barley meal | 10. | 57. |
| Rye meal | 10. | 57. |
| White potatoes | 10. | 86. |
| Blue potatoes | 10. | 115. |
| Rice | 10. | 123. |
| Buckwheat meal | 10. | 130. |

* Read before the Philadelphia Society of Veterinary Medicine.

The above table I give because it represents the food we eat; but the only part of interest to us in this table is the veal and the beef. Veal is put at the head of the list because it is of the poorest quality, showing 10 parts of nitrogenous to only 1 part of non-nitrogenous matter, while beef contains 10 parts nitrogenous and 17 parts of non-nitrogenous material. Hence you see the older veal is, the nearer it comes to the proper proportions of requirements to health.

The appearance of veal depends upon its size, to a certain extent, which should be well formed in proportion to the bone and fleshed up well on the ribs. The color should be of a reddish white; the fat white and bright, especially on the kidney, and the caul or Omentum should be heavy with fat.

Monkey veal is small, lank, and lean, the bone is small, thin on the ribs, reddish-blue in appearance, and has very little fat on the caul and kidney; the fat also has a blue appearance, the caul is thin and light. The carcass has a slimy, slippery look.

The above applies when they are very young. The caul and the kidney are the first to lay on fat that shows; it gradually becomes heavy and thick with fat as the animal grows older and takes on flesh. No young animal has fat in its tissues at birth.

The law regulating the matter in Philadelphia, is that a calf shall weigh 55 lbs., and must be three weeks old before it can be put on the market. In my opinion the above law will not properly protect against the sale of monkey veal; I have known many calves only three to ten days old which would weigh 90 to 110 lbs., and these would dress 50 lbs. to 65 lbs. Such calves come from a large breed of cattle, as the Durhams and Holsteins. I have bought calves of Durham stock which have weighed 109 lbs. when four days old. On the other hand take small cattle, as the Alderney with her first calf, which may be three weeks old and would not dress 50 lbs.; as Alderneys have been known to calve at 13½ months old. This calf must be small, as the mother is only a calf herself.

There is only one way to arrive at the result, and that is the appearance on inspection, after the calf has been slaughtered and dressed. If the inspector confiscates the carcass and prosecutes the offender, more care will be taken to see that the calves are old enough before they are killed. The butcher can tell by its looks, when alive, whether it is fat or not, and old enough.

Monkeys are not killed and sold without the butcher knowing it. They do it for gain, and the ignorant housekeeper buys it because it is cheap, or because he has a small family, and wants a small roast or cutlets, and he has to suffer with pain and diarrhœa.

The time when calves should be killed for food must be at a period when the most poorly nourished calf will be fit for food. One that has been well fed and pushed to gain flesh as fast as possible, does not meet the requirements The law should be changed to cover the first, which, I think, should be four weeks, and 65 lbs. in weight.

